# Dengue Hemorrhagic Fever Presenting With Pericardial Effusion and Cardiac Tamponade: A Case Report

**DOI:** 10.7759/cureus.76836

**Published:** 2025-01-03

**Authors:** Avinash Parepalli, Keyur Saboo, Sourya Acharya, Vijay Kota, Manikanta Nelakuditi

**Affiliations:** 1 Department of Medicine, Jawaharlal Nehru Medical College, Datta Meghe Institute of Higher Education and Research, Wardha, IND

**Keywords:** cardiac tamponade, cardiogenic shock, dengue, hemorrhagic fever, polyserositis

## Abstract

Dengue fever encompasses a spectrum of illnesses, ranging from mild dengue fever to more severe forms, including dengue hemorrhagic fever (DHF) and dengue shock syndrome (DSS). Cardiogenic shock and multi-organ failure are among the complications that can arise from the syndrome, which is considered to be caused by endothelial cell loss and increased capillary permeability. Its incidence is especially significant during monsoon seasons. A 45-year-old woman required intubation after presenting with hypotension and changed mental status after suffering from a high-grade fever, severe headache, myalgias, and progressive respiratory distress for seven days. An emergency was called when imaging indicated a considerable pericardial effusion, which echocardiography verified. Pericardiocentesis was carried out, and 300 mL of hemorrhagic fluid was collected. She then required inotropic therapy to avoid cardiogenic shock. After receiving empirical antibiotics, platelet transfusions, and close supervision, the patient's health improved, and the pericardial drain was removed on the fifth day of hospitalization. Since myocarditis and pericardial effusion are uncommon but hazardous cardiac symptoms in dengue fever, it is critical to detect and treat these disorders in patients with severe dengue fever to limit the illness's morbidity and mortality.

## Introduction

Dengue fever is a significant public health issue, particularly in tropical and subtropical countries, and is transmitted through the bite of Aedes aegypti mosquitoes [1]. Dengue virus is an RNA (ribonucleic acid) virus from the Flaviviridae family, with a single-stranded positive-sense RNA genome [2]. Four serotypes of dengue virus are well known worldwide (dengue virus-1, dengue virus-2, dengue virus-3, and dengue virus-4). Dengue infection can cause a variety of syndromes, including dengue fever, dengue hemorrhagic fever (DHF), and dengue shock syndrome (DSS). Dengue infection can cause complications such as liver failure, acute kidney injury, microangiopathic hemolytic anemia (including hemolytic uremic syndrome), disseminated intravascular coagulation, encephalopathy, and, in some cases, cardiac and neuromuscular dysfunctions [3,4].

The pathogenesis of the illness is the destruction of the endothelial cells and increased capillary leakage, which leads to reduced intravascular volume and the development of DSS or DHF [3,5]. Ultrasonography may determine the severity of dengue infection by detecting plasma leakage, featuring ascites, pleural effusion, perirenal fluid, subcapsular hepatic collection, and pericardial effusion [4]. One such fatal complication due to dengue infection is the development of pericardial effusion, which can progress to cardiac tamponade. Cardiac tamponade can lead to hemodynamic instability and requires immediate intervention. Some researchers have also reported that the development of shock in dengue infection is due to cardiac dysfunction, such as pericarditis and myocarditis, directly caused by the virus [3,4].

A 2012-2014 study of 150 dengue patients from Tamil Nadu, India, discovered that the majority of infections occurred during and after the monsoon season. Fifty-four percent of the participants were middle-aged, between 21 and 40 years old. The most common symptoms were fever, followed by lethargy (81.3%) and myalgia (60.7%). Warning signals were detected by 22.7%, with 19.3% being diagnosed with severe dengue, along with acute renal damage (7.3%), myocarditis (1.3%), and encephalopathy (1.3%). Comorbid diseases were present in 10% of patients, and they were substantially related to severe dengue. A laboratory examination indicated the presence of leucopenia at 4% and serositis at 15.3%. No fatalities were reported, demonstrating the effectiveness of treatment and care [6].

The following case report outlines a 45-year-old female patient who suffered hemorrhagic pericardial tamponade as a result of dengue infection.

## Case presentation

A 45-year-old woman presented to the emergency room with a seven-day history of high fever, severe headache, and myalgias. The patient initially developed a fever, which was high grade and associated with severe headache retroorbital pain, and generalized muscle pains. Two days after the onset of the fever, the patient developed a rash that began as a painless erythematous macule over the flexor aspects of the lower limbs, which progressed to the upper limbs in two days. The patient also complained of generalized weakness, and she was lethargic; additionally, patient complaints of chest discomfort and heaviness progressed in the course of the disease. Later, the patient developed shortness of breath during slight exertion, which progressed such that it worsened in two days to dyspnea at rest. Alongside these symptoms, the patient also developed bilateral lower limb swelling. The patient had no prior history of comparable symptoms, no previous operations, no continuing treatment history, and no history of substance misuse or exposure.

Upon assessment, the patient was febrile (102°F), tachycardic (110 beats per minute), and exhibited pulse abnormalities (pulse paradoxes). She was hypotensive and in shock (blood pressure: 80 systolic), tachypneic, had a respiratory rate of 25 per minute, and had 86% saturation on pulse oximetry at room air. On inspection, the patient appears lethargic and pale; her jugular venous pressure was elevated with rapid y descent and was measured to be 12 cm; palpation of the abdomen reveals tender hepatomegaly with mild right upper quadrant tenderness; and on auscultation of the heart, muffled heart sounds with s3 gallop and pericardial rub was noted. Her GCS (Glasgow Coma Scale) was 5/15; therefore, she was intubated and placed on mechanical ventilator support.

**Figure 1:**
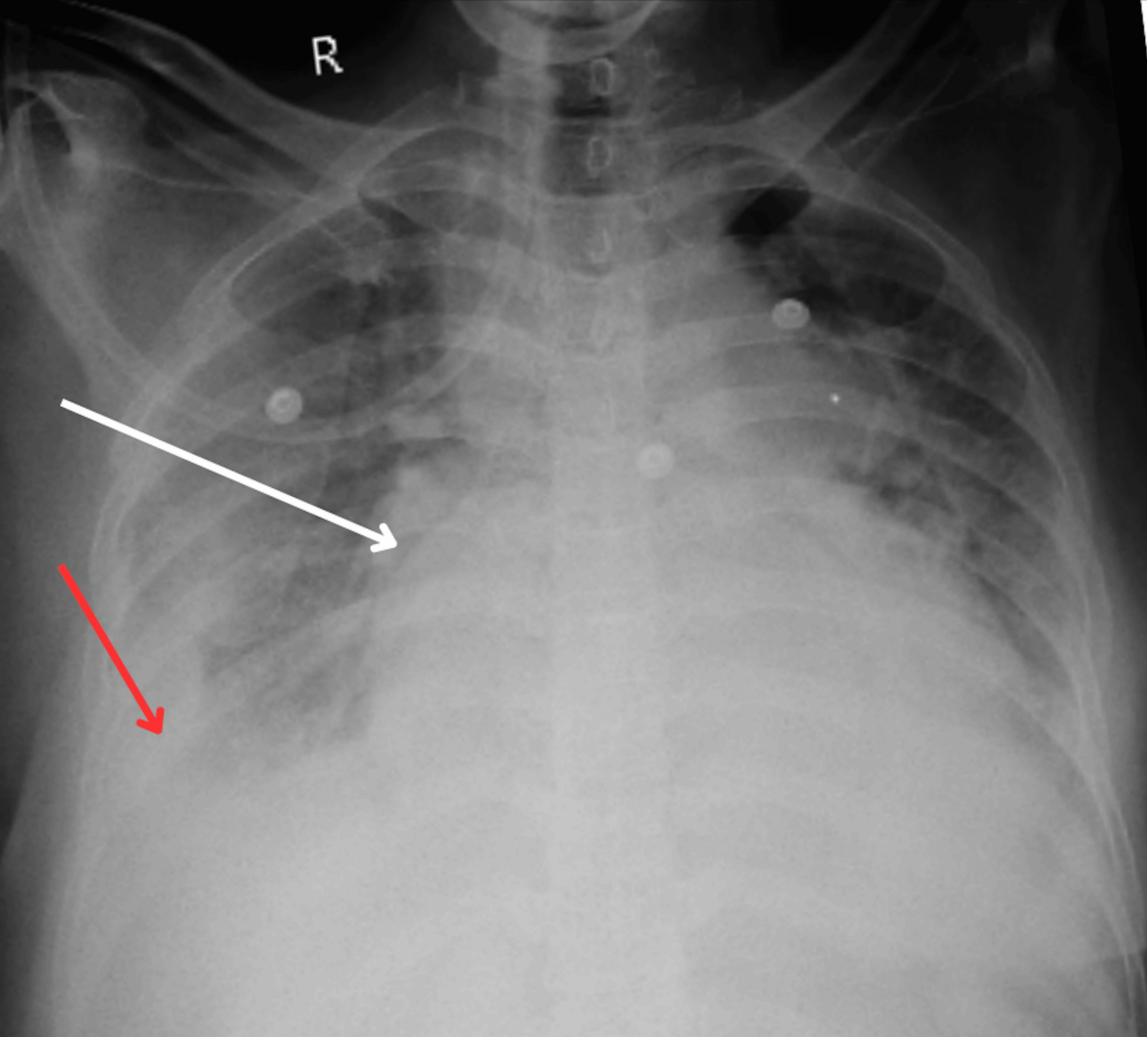
Chest X-ray showing pericardial effusion (white arrow) and pleural effusion (red arrow)

Chest X-ray, as shown in Figure 1, showed globular enlargement of the cardiac shadow with a widening of the subcarinal angle and differential density sign at the cardiac border, which could be due to pericardial effusion or cardiomegaly (suggest 2Decho correlation). Blunting of bilateral costophrenic angles is suggestive of pleural effusion.

**Figure 2:**
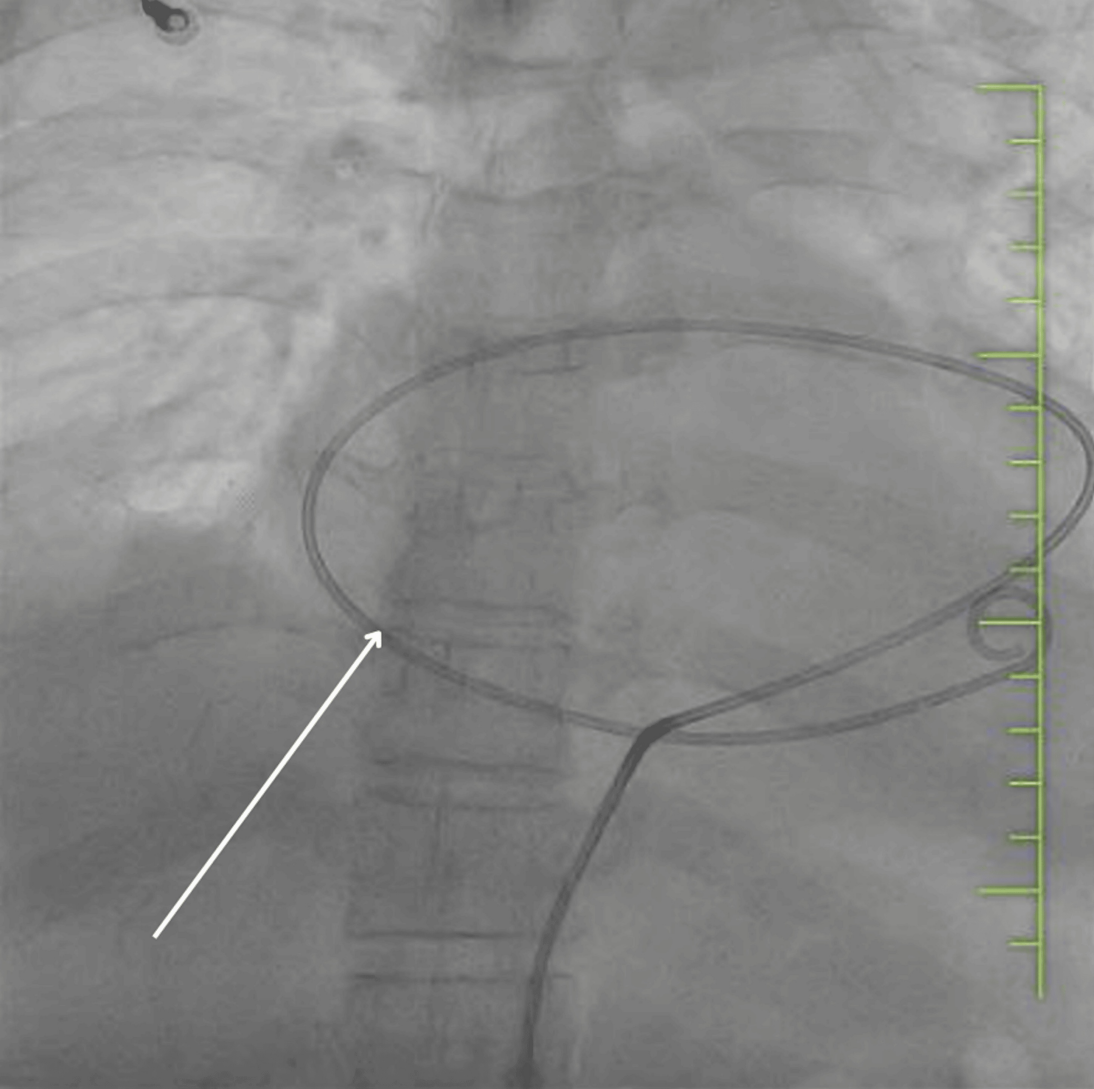
Chest radiograph with pericardial drain indicated by white arrow

A chest ultrasound was performed, revealing massive pericardial effusion with signs of pericardial tamponade. The patient was then transferred to the cath lab, where an emergency pericardiocentesis was performed, and a pericardial drain was placed (see Figure 2). A total of 300 mL of pericardial fluid was drained.

**Figure 3:**
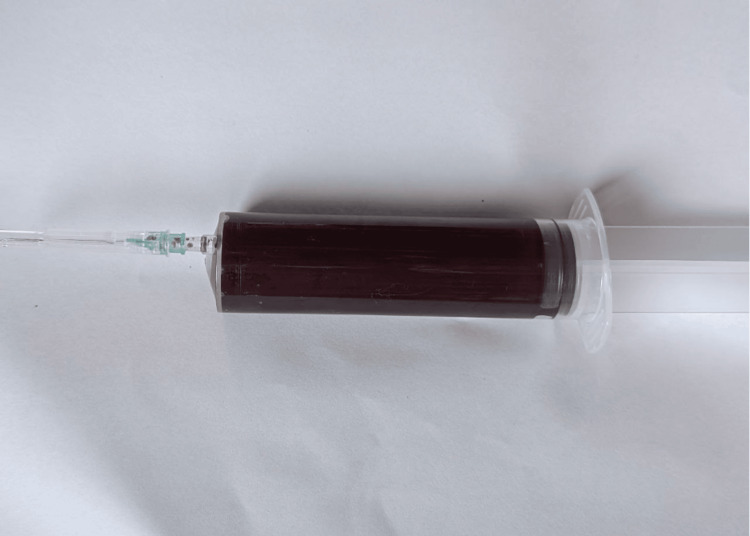
Pericardial fluid: red and hemorrhagic in nature

The pericardial fluid was red and hemorrhagic, as seen in Figure 3, indicating the presence of blood within the pericardial sac.

Given the cardiogenic shock due to cardiac tamponade, inotropic support was provided with a continuous intravenous infusion of noradrenaline at 0.4 mcg/kg/min. Routine laboratory investigations and a fever profile were also obtained (Table 1).

**Table 1:** Lab parameters of the patient

Lab Parameters	Observed Value	Normal Range
Hb (Hemoglobin)	8.7 gm%	13-17 gm%
MCV (Mean Corpuscular Volume)	72 fL	83-101 fL
TLC (Total Leucocytic Count)	2200 cells/cu mm	4000-10000 cells/cu mm
Neutrophils	84%	40-60%
Lymphocytes	11%	20-40%
Monocytes	03%	2-8%
Basophils	00%	0.5-1%
Eosinophils	02%	1-4%
Platelets	0.15 lakhs/cu mm	1.5-4.1 lakhs/cu mm
Urea	28 mg/dL	19-43 mg/dL
Creatinine	1.2 mg/dL	0.66-1.25 mg/dL
Sodium	136 mmol/L	137-145 mmol/L
Potassium	4.3 mmol/L	3.5-5.1 mmol/L
Calcium	8.9 mg/dL	8.4-10.2 mg/dL
Magnesium	2.0 mg/dL	1.6-2.3 mg/dL
Phosphorous	2.9 mg/dL	2.5-4.5 mg/dL
Uric Acid	3.9 mg/dL	3.5-8.5 mg/dL
Alkaline Phosphatase	92 U/L	38-126 U/L
ALT (Alanine Transaminase)	86 U/L	<50 U/L
AST (Aspartate Transaminase)	73 U/L	17-59 U/L
Albumin	3.2 g/dL	3.5-5 g/dL
Total Bilirubin	0.9 mg/dL	0.2-1.3 mg/dL
Conjugated Bilirubin	0.3 mg/dL	0.0-0.3 mg/dL
Unconjugated Bilirubin	0.6 mg/dL	0.0-1.1 mg/dL
RBS (Random Blood Sugar)	110 g/dL	90-140 g/dL
LDH	379 U/L	140-280 U/L

**Figure 4:**
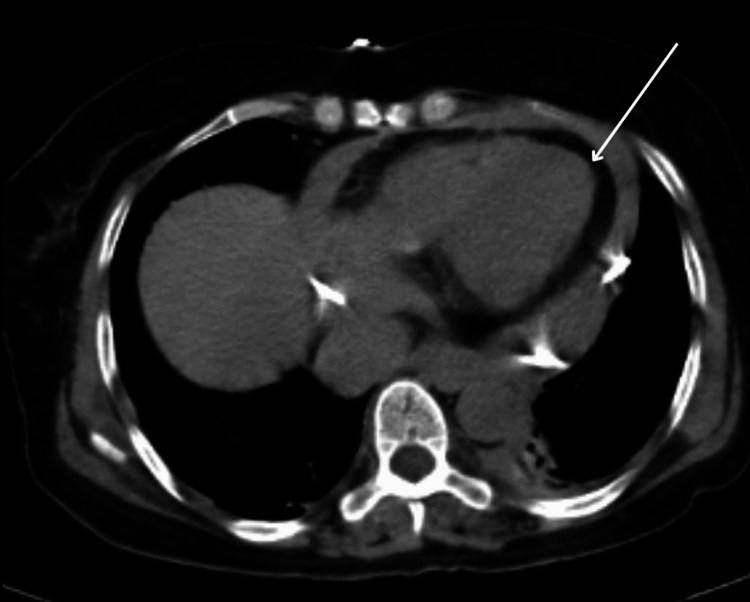
High-resolution CT showing pericardial effusion, indicated by the white arrow CT, computed tomography

The patient was started on empirical antibiotics, including injectable doxycycline, to prevent secondary infections, as well as injectable corticosteroids. Additionally, the patient was transfused with 5 units of random donor platelets due to a low platelet count. Adequate hydration was maintained. Daily input-output monitoring, regular blood pressure and SpO₂ monitoring, and random blood sugar charting were performed, with symptomatic treatment provided as needed. A high-resolution computed tomography (CT) scan of the patient revealed minimal left-sided pleural effusion (approximately 150 mL) with basal atelectasis. Gross pericardial effusion was noted, with a maximum thickness of 18 mm and a drain in situ, as shown in Figure 4.

The patient's pericardial fluid was drained daily under aseptic conditions, and symptomatic and supportive treatments such as intravenous fluids, antiemetics, antipyretics, and proton pump inhibitors were administered. The patient's health progressively improved, and the pericardial fluid began to drain slowly. The pericardial drain was removed on day five of hospitalization, and the patient was treated conservatively with diuretics. The patient's health improved throughout therapy, and she was discharged on medicine with the recommendation for further follow-up.

## Discussion

Cardiac symptoms are common in DHF, with involvement that can range from benign bradycardia to severe myocarditis as well as cardiac tamponade, as in this example. Cardiac symptoms of DENV infection are uncommon but common in severe patients [3]. A study of 6,773 people indicated that sinus bradycardia was the most common cardiac abnormality (8.8%), whereas myocarditis and pericarditis were seen in 2.9% and 0.1% of instances, respectively [4]. Uncommon cardiac symptoms are more prevalent in persons suffering from severe and non-severe dengue with warning signals compared to patients with non-severe dengue without warning signs [5].

Dengue myocarditis/pericarditis is exceedingly rare, with just a few cases reported in the literature across the globe. Perimyocarditis as well as myopericarditis are not frequently reported in DENV infections, with the latter having a low normal ejection fraction [6,7].

In research that detailed pathology findings in 100 fatal dengue patients, the two major gross findings were widespread mucosal bleeding and serous membrane edema [8]. Gross hemorrhages were evident in several organs, and microscopic investigation revealed hemorrhages as well as perivascular and interstitial tissue edema. In addition, several patients showed mononuclear infiltration surrounding arteries as well as endothelial cell pyknosis. Later research discovered the presence of dengue antigen in several cell types, namely monocytes, Kupffer cells, alveolar macrophages, peripheral blood and splenic lymphocytes, and endothelial cells of the liver and lung [1,9].

Myocarditis in DHF is usually mild, although it can be deadly on occasion. It is assumed to be the result of cytokine-induced myocardial injury. Direct infiltration of cardiac tissue by the dengue virus may be part of this process, although it is uncertain. Pericardial effusion in DHF is often minor and not clinically significant, occurring as a result of serositis. Setiawan et al. [4] discovered that substantial pericardial effusion occurred in only 8% of severe dengue patients. Life-threatening cardiac tamponade induced by rapidly accumulating pericardial effusion is relatively uncommon. The proposed mechanism is cytokine-mediated endothelial dysfunction, which eventually results in increased vascular permeability and third-space fluid loss [5,10].

Several research studies indicated that immature dendritic cells may become infected with DENV, causing them to activate and develop [11]. A number of studies utilizing skin explants have demonstrated that DCs in the skin can get infected with locally inoculated DENV. The risk of DHF rises with successive infections; it has been proposed that pre-existing cross-reactive, non-neutralizing antibodies promote viral absorption by host cells, hence enhancing replication. According to several studies, DHF patients had a greater viral load than dengue fever patients [12]. Furthermore, circulating levels of NS1 protein were greater in individuals with more severe illnesses. Most elements of DENV do not support the production of endothelial cell death. However, studies have revealed that the soluble version of the NS1 protein activates complement, which might lead to plasma leakage [10,12].

The dynamics of viral load, which rises during presentation (febrile phase) and falls substantially during defervescence and plasma leakage, do not appear to support the virus's direct impact on vascular permeability. DENV-infected cells produce proinflammatory cytokines such as TNF-α, IL-6, IL-8, and chemokines, which may indirectly lead to increased permeability. Such mediators have been found at high levels in DHF patients.

Though less usually documented, cardiac tamponade in DHF is important to a correct diagnosis. Patients with DHF who experience hypotension, shock, or respiratory distress may have increased vascular permeability and "plasma leak." In such circumstances, therapy is mostly supportive, with continuous monitoring of the patient. In this situation, clinical deterioration and mild effusion are insufficient to explain severe shock. Cardiac dysfunction signs such as elevated jugular venous pressure, muffled heart sounds, and low blood pressure indicated a probable cardiac tamponade, which was verified with echocardiography [13,14]. Elevated troponin-I alterations on an ECG suggested myopericarditis. Echocardiography revealed no regional or global wall motion anomalies, and the ejection fraction was normal. So, the odds of myocardial ischemia were decreased [5,12,14].

## Conclusions

Through the example of a 45-year-old woman who acquired severe DHF, which resulted in major cardiac problems such as pericardial effusion and cardiac tamponade, this study highlights the dangerous sequelae associated with dengue fever. This example demonstrates how an illness such as dengue can develop from feverish symptoms to serious consequences, highlighting the urgent need for early diagnosis and treatment. The findings just serve to confirm that, even if cardiac symptoms are uncommon in dengue patients, they might manifest as serious illnesses like myocarditis and cardiac tamponade, particularly in cases of DHF. Ultrasonography monitoring was crucial for determining the severity and successfully handling problems. The patient's positive response to treatment, including pericardiocentesis and inotropic support, underscores the necessity of timely interventions to prevent deterioration. This case contributes to the existing literature indicating that dengue can lead to significant cardiac complications and calls for heightened awareness among providers in regions where dengue is endemic. Subsequent studies should explore mechanisms of injury to better outcomes in critically ill patients with severe dengue infections.
